# Prevalence and Characterization of Carbapenem-Resistant Enterobacteriaceae Isolated from Mulago National Referral Hospital, Uganda

**DOI:** 10.1371/journal.pone.0135745

**Published:** 2015-08-18

**Authors:** Deogratius Okoche, Benon B. Asiimwe, Fred Ashaba Katabazi, Laban Kato, Christine F. Najjuka

**Affiliations:** Department of Medical Microbiology, Makerere University College of Health Sciences, Kampala, Uganda; Iowa State University, UNITED STATES

## Abstract

**Introduction:**

Carbapenemases have increasingly been reported in enterobacteriaceae worldwide. Most carbapenemases are plasmid encoded hence resistance can easily spread. Carbapenem-resistant enterobacteriaceae are reported to cause mortality in up to 50% of patients who acquire bloodstream infections. We set out to determine the burden of carbapenem resistance as well as establish genes encoding for carbapenemases in enterobacteriaceae clinical isolates obtained from Mulago National Referral Hospital, Uganda.

**Methods:**

This was a cross-sectional study with a total of 196 clinical isolates previously collected from pus swabs, urine, blood, sputum, tracheal aspirates, cervical swabs, endomentrial aspirates, rectal swabs, Vaginal swabs, ear swabs, products of conception, wound biopsy and amniotic fluid. All isolates were subjected to phenotypic carbapenemase screening using Boronic acid-based inhibition, Modified Hodge and EDTA double combined disk test. In addition, all the isolates were subjected to PCR assay to confirm presence of carbapenemase encoding genes.

**Results:**

The study found carbapenemase prevalence of 22.4% (44/196) in the isolates using phenotypic tests, with the genotypic prevalence slightly higher at 28.6% (56/196). Over all, the most prevalent gene was *bla*VIM (21,10.7%), followed by *bla*OXA-48 (19, 9.7%), *bla*IMP (12, 6.1%), *bla*KPC (10, 5.1%) and *bla*NDM-1 (5, 2.6%). Among 56 isolates positive for 67 carbapenemase encoding genes, *Klebsiella pneumonia* was the species with the highest number (52.2%). Most 32/67(47.7%) of these resistance genes were in bacteria isolated from pus swabs.

**Conclusion:**

There is a high prevalence of carbapenemases and carbapenem-resistance encoding genes among third generation cephalosporins resistant Enterobacteriaceae in Uganda, indicating a danger of limited treatment options in this setting in the near future.

## Introduction

Enterobacteriaceae is a family of gram-negative bacteria and members are mainly inhabitants of the gut flora [1,2]. Most members of this family are pathogenic and cause such human infections as gastrointestinal infections, septicemia, pneumonia, meningitis, peritonitis and urinary tract infections [2,3]. These organisms easily acquire and transfer drug resistance genes through plasmids and transposons. Acquisition of these genes leads to production of β-lactamases of which extended spectrum β-lactamases (ESBLs) are the most common [3]. ESBLs in enterobacteriaceae are reported to coexist with resistance to other antimicrobial classes and as such these organisms become multi-drug resistant hence limiting treatment options for infections. Carbapenem antibiotics have been used as a last resort to treat infections caused by multidrug resistant gram negative bacteria [4,5]. However, there has been emergence of carbapenem resistant bacteria which now have a worldwide presence [2], thought to be due to high antibiotic use and misuse without proper diagnosis of infection, or self-medication by patients [6]. As a result, there is selective pressure on microorganisms, which in turn enhances antimicrobial resistance. Infections caused by bacteria resistant to carbapenems often fail to respond to conventional treatment, and are said to kill up to 50% of patients with bloodstream infection due to them [2,7].

Resistance to carbapenems is mostly due to the production of carbapenemases, which are *β*-lactamase enzymes with a capacity to hydrolyze not only the carbapenems but also all the other beta lactam agents [8]. The most common carbapenemases include veronica integron metallo-beta-lactamases types (VIM), imipenemase (IMP) types, *Klebsiella pneumoniae* carbapenemase (KPC), oxacillinase-48 (OXA-48), and New Delhi metallo-beta-lactamase-1 (NDM-1), encoded by carbapenem resistance determining genes *bla*VIM, *bla*IMP, *bla*KPC, *bla*OXA-48, and *bla*NDM, respectively [2]. Phenotypic assays are used to identify carbapenemase activity while molecular assays have been developed to identify carbapenemase encoding genes [2,9].

In most sub-Saharan Africa, there is limited data on the prevalence and distribution of carbapenem resistance among enterobacteriaceae. In East Africa, a few studies have been done in Kenya and Tanzania. A surveillance study in Kenya reported isolation of NDM-1 producing *Klebsiella pneumoniae* from urine samples [10], while in Tanzania a study reported a prevalence of 35.24% carbapenemase genes among multi-drug resistant gram-negative bacteria based the PCR assays [5]. Isolation of carbapenemase producers among ESBL isolates was also reported in South Africa [11,12]. In Uganda, however, no documented study has previously been done to ascertain the magnitude of carbapenemase producers in health care or community settings. This study set out to determine the prevalence of carbapenemases and carbapenemase encoding genes among clinical enterobacteriaceae obtained from patients at the National Referral and Hospital, Mulago.

## Methods

### Study design and population

This was a cross-sectional laboratory based study, between January, 2013 and March, 2014 inclusive, involving 196 stored enterobacterial clinical isolates resistant to at least two third generation cephalosporins. We chose this ample because carbapenemase producers are virtually resistant to all third generation cephalosporins [13] and hence provide the most sensitive indicator of carbapenemase production [14]. The clinical specimens were previously obtained from patients referred by Mulago hospital to the microbiology laboratory, of the Department of Medical Microbiology, Makerere University College of Health Sciences.

### Controls used in the study

For quality control, well characterized strains were used. *E*. *coli* ATCC 25922 was used as a susceptible strain, *Klebsiella pneumoniae* ATCC BAA-1705 as a positive control while *Klebsiella pneumoniae* ATCC BAA-1706 was used as a negative control. These control strains were obtained from Microbiologic, MediMark (Grenole Cedex2, France). For EDTA, Boronic acid and PCR tests, the following control strains were used; *E*. *cloacae* JMI10526 for *bla*IMP, *Acinetobacter baumannii* AB5 for *bla*VIM, *K*. *pneumoniae* ATCC strain BAA-1705 for *bla*KPC, *K*. *pneumoniae* ATCC strain BAA-2146 for *bla*NDM-1, and *E*. *coli* ATCC BAA-2523 for *bla*OXA-48.

### Recovery of study isolates

We characterized 196 previously stored multidrug resistant enterobacterial isolates. Isolates were sub-cultured on blood agar from the stock collection and incubated in ambient air at 35°C overnight to obtain fresh bacterial growth. All the isolates were re-identified using API 20E (BioMerieux, Inc., Hazelwood, MO). The number of isolates obtained from various clinical specimens is shown in Table 1.

**Table 1:** Number of isolates obtained from various clinical specimens.

Species	SPECIMEN													
	Blood	Cervical swab	Ear swab	Endomentrial aspirate	Product of conception	Pus swab	Rectal swab	Sputum	Tracheal aspirate	urine	Vaginal swab	Wound biopsy	Amniotic fluid	Total
*E*. *coli*	8	3	2	2	1	25	2	3	2	35	0	1	0	84
*Enterobacter Spp*	1	1	0	0	0	5	0	0	0	2	0	0	0	9
*Klebsiella oxytoca*	0	0	0	0	0	1	0	0	0	3	0	0	0	4
*Klebsiella pneumoniae*	4	3	0	3	0	30	0	8	8	20	2	0	0	78
*Pantoea agglomerans*	0	0	0	0	0	1	0	0	0	0	0	0	0	1
*Proteus mirabilis*	0	0	0	0	0	2	0	0	1	3	0	0	0	6
*Proteus vulgaris*	0	0	0	0	0	1	0	0	1	1	0	0	0	3
*Salmonella spp*	1	0	0	0	0	0	0	0	0	0	0	0	0	1
*Serratia marcescens*	0	0	0	0	0	2	0	0	0	0	0	0	0	2
*Citrobacter freundii*	0	0	0	0	0	3	2	0	0	2	0	0	1	8
**Total**	14	7	2	5	1	70	4	11	11	67	2	1	1	**196**

### Susceptibility testing

Susceptibility testing was done for the following10 antibiotics: Amoxycillin clavulanate (AMC) (30μg); Cefuroxime (CXM)(30μg); Temocillin(TEM)(30μg); Piperacillin-tazobactum (TPZ) (110μg); Cefoxitin (FOX)(30μg); Cefipime (FEP)(30μg); Ceftriaxone (CRO)(30μg); Ceftazidime (CAZ) (30μg); Cefotaxime(CTX)(30μg); Meropenem (MEM)(10μg).

This was done using the Kirby Bauer disk diffusion, and interpreted according to Clinical and Laboratory Standards Institute (CLSI) guidelines [14] in order to find out if there was a relation between resistance to these drugs and carriage of carbapemenases, as previously reported elsewhere [5,15].

### Detection of carbapenemase production

The Modified Hodge test (MHT), Boronic acid synergy test and the EDTA for the detection of carbapenemase production were performed as described elsewhere [2,9,14]. Briefly, in the MHT, a 1:10 dilution of the *E*.*coli* ATCC 25922 was made by adding 0.5 ml of the 0.5 McFarland to 4.5 ml of saline which was then streaked all over the plate using a swab. Thereafter, 10 μg meropenem disk (BiolabZrt, Budapest, Hungary) was placed in the center of the test area. The test isolate was streaked in a straight line from the disk to the edge of the plate. Interpretation of both negative and positive tests was done according to CLSI [14]. The Boronic acid synergy test was done by adjusting the inoculum to a 0.5 McFarland turbidity standard and then streaked on a plate by swabbing. The disks of 10μg meropenem and 400μg of phenylboronic Acid (PBA) (BiolabZrt, Budapest, Hungary) were then placed on the inoculated plate 15mm apart center to center, and incubated for 24 hours. The plate was then examined for the presence of an enhanced growth inhibition zone between the carbapenem disk and Boronic acid disk. The test with an enhanced growth inhibition zone was considered positive for the detection of KPC enzyme production, as described elsewhere [16,17]. For the Ethylenediammine tetra acetic acid (EDTA) test, an overnight liquid culture of the test isolate was adjusted to a turbidity of 0.5 McFarland standard and spread on the surface of a Mueller Hinton Agar plate. Two 10 μg imipenem discs were placed on the agar 15 mm apart (center to center). 10 μl of 0.5 M EDTA was added to one of the imipenem disc to get a desired concentration of 750 μg [18]. After incubation at 37°C overnight, increase of inhibition zone diameter of more ≥5mm in the disc potentiated with the EDTA was interpreted as positive for metallo-β-lactamase production as described elsewhere [18,19].

### Detection of carbapenemase encoding genes

Polymerase chain reaction (PCR) was carried in a TC-412 thermocycler machine (BBC Scientific Ltd, UK). The genes *bla*IMP and *bla*VIM were amplified using primers and conditions as described in the Tanzania study [5]; while genes *bla*KPC, *bla*OXA-48 and *bla*NDM-1 were amplified using primers and conditions as described by Karuniawati *et al* [6]. Isolates that were positive for boronic acid test but negative for blaKPC were further tested for six AmpC genes: DHA, EBC, MOX, FOX, CIT, and ACC to see if ampC genes were responsible for false positive from boronic acid test. Amplicons were analysed by gel electrophoresis in 1.5% agarose and documented using a bioimager.

### Data analysis

Data analysis was done in STATA version 12. Out comes were presented as proportions and percentages in a tabular form. A *P*-value of ≤ 0.05 was considered as evidence of significant statistical difference.

### Ethical issues

This study was approved by the Institutional Review Board of the School of Biomedical Sciences, Makerere University College of Health Sciences; and the board waived the need for consent to use the archived isolates.

## Results

### Socio-demographics and isolate characteristics

The study used 196 stored clinical isolates that were obtained from 102 male and 94 female patients. The median age of the participants was 30 years, with a range of 1 to 86 years. All the 196 Enterobacteriaceae isolates were reconfirmed to be resistant to at least two of the three third generation cephalosporins tested (ceftriaxone, ceftazidime and cefotaxime). Of the 196 isolates, 82 were *E*. *coli*, 78 *Klebsiella pneumoniae*, 11 *Enterobacter spp*, eight for *Citrobacter freundii*, six for *Proteus mirabilis*, four for *Klebsiella oxytoca*, three for *Proteus vulgaris*, two for *Serratia marcescens*, and one each for *Pantoea agglomerans* and *Salmonella spp* (Table 1).

### Susceptibility to antibiotics

For antibiotic susceptibility testing, 36/196 (18.4%) study isolates were resistant to meropenem according to CLSI interpretation, of which 16/36 were positive for carriage of one or more carbapenemase gene by PCR. However, out of the 160 clinical isolates that were sensitive to meropenem, 40 were positive for carriage of one or more carbapenemase gene by PCR. However when these 40 isolates were tested with ertapenem and imipenem, all were sensitive to imipenem, while only 1 was sensitive, 5 intermediate and 34 resistant to ertapenem. Similarly, Out of 106 study isolates with temocillin diameters of ≥12mm and piperacillin/tazobactam diameters ≥16 mm, eight were positive for *bla*OXA-48 gene when subjected to PCR. Furthermore, 11 of 36 isolates with temocillin zone diameter of <12mm had the *bla*OXA-48 gene as determined by PCR. The full antibiogram for the tested drugs is seen in Table 2.

**Table 2:** Susceptibilitypattern of bacteria used in the study.

Species	AMC	CXM	TEM	TPZ	FOX	FEP	CRO	CAZ	CTX	MEM
*Citrobacter freundii*(n = 8)	0.0%	0.0%	25.0%	0.0%	37.5%	12.5%	0.0%	12.5%	0.0%	87.5`%
*`Enterobacter spp*.(n = 9)	0.0%	0.0%	33.3%	11.1%	11.1%	0.0%	0.0%	0.0%	0.0%	44.4%
*E*. *coli* (n = 84)	0.0%	1.2%	8.3%	23.8%	32.1%	1.2%	0.0%	0.0%	0.0%	90.5%
*Klebsiella oxytoca*(n = 4)	25.0%	0.0%	0.0%	25.0%	25.0%	0.0%	0.0%	25.0%	0.0%	100%
*Klebsiella pneumoniae*(n = 78)	1.3%	2.6%	16.7%	23.1%	41.0%	3.8%	0.0%	0.0%	0.0%	74.4%
*Pantoeaag glomerans* (n = 1)	0.0%	0.0%	0.0%	0.0%	0.0%	0.0%	0.0%	0.0%	0.0%	100%
*Proteus mirabilis* (n = 6)	0.00%	0.0%	0.0%	50.0%	16.7%	0.0%	0.0%	0.0%	0.0%	83.3%
*Proteus vulgaris*(n = 3)	0.0%	0.0%	33.3%	66.7%	0.0%	0.0%	0.0%	0.0%	0.0%	100%
*Salmonella spp*.(n = 1)	0.0%	0.0%	0.0%	0.0%	0.0%	0.0%	0.0%	0.0%	0.0%	100%
*Serratia marcescens* (n = 2)	0.0%	0.0%	0.0%	0.0%	0.0%	0.0%	0.0%	0.0%	0.0%	50.0%

AMC-Amoxycillin clavulanate

SXT-Trimethoprimsulfamethoxazole

CXM-Cefuroxime

TEM-Temocillin

TPZ-tazobactumpipperacillin

FOX-Cefoxitin

FEP-Cefipime

CRO-Ceftriaxone

CAZ-Ceftazidime

CTX-Cefotaxime

MEM-Meropenem.

### Prevalence of carbapenemase activity based on phenotypic tests

Carbapenamase activity was detected in 20/196 (10.2%) by MHT method, 22/196 (11.2%) by Boronic acid screen and 7/196 (3.6%) by the EDTA test. Five of the isolates were positive for both the MHT and Boronic acid methods. Overall, 44 (22.4%) of the 196 clinical isolates were positive for the production of one or more carbapenemases. Details of the carbapenemase activity among the isolates are shown in Table 3.

**Table 3:** Bacterial isolates positive for a particular phenotypic test.

Species	Number of isolates positive for a particular phenotypic test				
	MHT	Boronic acid (APB)	MHT and Boronic positive	MHT positive and Boronic negative	EDTA
*Citrobacterfreundii*	1	0	0	1	0
*E*. *coli*	9	9	2	7	2
*Enterobacter spp*.	2	1	0	2	2
*Klebsiella oxytoca*	1	1	1	0	0
*Klebsiella pneumoniae*	4	11	2	2	2
*Pantoeaagglomerans*	0	0	0	0	1
*Proteus mirabilis*	2	0	0	2	0
*Proteus vulgaris*	1	0	0	1	0
*Salmonella spp*	0	0	0	0	0
*Serratiamarcescens*	0	0	0	0	0
**Total**	**20**	**22**	**5**	**15**	**7**

MHT positive = *KPC* +*OXA*48

Boronic acid positive = *KPC*

MHT + Boronic acidpositive = *KPC*

MHT positive + boronic acid negative = *OXA* 48

EDTA positive = Metallo-β-lactamase.

### Prevalence and distribution of Carbapenemase genes

Based on the PCR assays, 56/196 (28.6%) of the isolates were positive for one or more carbapenemase genes. We confirmed the PCR products for the genes detected using different restriction enzymes, and the fragments obtained were of the expected sizes. *Klebsiella pneumonia*e was the species with the highest number of these genes. Eight of the 56 (14.3%) carbapenemase gene carrying isolates harbored two or more genes. Multiple genes were only carried in *Klebsiella pneumoniae*, *Enterobacter spp*, *Proteus mirabilis* and *E*.*coli* (Table 4). Plasmid DNA was extractedfrom these eight isolates and tested for carriage of carbepenemase genes. Only two of these isolates carried genes in their plasmid: one had *bla*OXA-48 and *bla*NDM-1 and the other had *bla*OXA-48 and *bla*VIM. Over all, the most prevalent gene was *bla*VIM 10.7%, followed by *bla*OXA-48 9.7%, *bla*IMP 6.1%, *bla*KPC 5.1% and *bla*NDM-1 2.6%. The genes were unevenly distributed among the different study isolates. A total of 67 carbapenemase encoding genes were found in the 196 isolates. *Klebsiella pneumoniae* had the highest number of these genes at 35 (52.2%), followed by *E*. *coli* (28.4%, n = 19), *Enterobacter spp* (7.5%, n = 5), *Serratia marcescens* (4.5%, n = 3), *Proteus mirabilis* had (3.0%, n = 2), *Citrobacter freundii*, *Klebsiella oxytoca*, and *Pantoea agglomerans* each each with one isolate(1.5%). No carbapenemase-ecoding genes were detected from *Proteus vulgaris* and *Salmonella spp* (Table 5).

**Table 4:** The number of bacteria positive for carbapenemase genes and number of genes per organism.

Species	Number of isolates	Genes per isolate	Genes present
*Klebsiella pneumoniae*	1	3	NDM-1, VIM, and KPC
*Enterobacter spp*.	1	3	VIM, KPC, and OXA-48
*Klebsiella pneumoniae*	1	3	VIM, KPC, and OXA-48
*Klebsiella pneumoniae*	2	2	VIM and OXA-48
*Klebsiella pneumoniae*	1	2	IMP and OXA-48
*Proteus mirabilis*	1	2	KPC and OXA-48
*E*. *coli*	1	2	KPC and OXA-48
*Klebsiella pneumoniae*	23	1	NDM-1 or VIM or IMP or OXA-48 or KPC
*Enterobacter spp*.	2	1	NDM-1 or VIM or OXA-48 or KPC
*E*. *coli*	17	1	VIM or IMP or OXA-48 or KPC
*Citrobacter freundii*	1	1	VIM
*Klebsiella oxytoca*	1	1	OXA-48
*Pantoea agglomerans*	1	1	IMP
*Serratia marcescens*	3	1	VIM or KPC

**Table 5:** The distribution of carbapenemase encoding genes in the study isolates.

Species	Carbapenemase-encoding genes					
	*bla*NDM-1	*bla*VIM	*bla*IMP	*bla*KPC	*bla*OXA-48	Total genes
*Citrobacter freundii* (n = 8)	0	1	0	0	0	1
*E*. *coli* (n = 84)	0	1	6	4	8	19
*Enterobacter spp*.(n = 9)	1	1	0	1	2	5
*Klebsiella oxytoca* (n = 4)	0	0	0	0	1	1
*Klebsiella pneumoniae* (n = 78)	4	16	5	3	7	35
*Pantoea agglomerans* (n = 1)	0	0	1	0	0	1
*Proteus mirabilis* (n = 6)	0	0	0	1	1	2
*Proteus vulgaris* (n = 3)	0	0	0	0	0	0
*Salmonella spp* (n = 1)	0	0	0	0	0	0
*Serratia marcescens* (n = 2)	0	2	0	1	0	3
**Total**	5	21	12	10	19	**67**

### Correlation of the phenotype and genotype of carbapenem resistance

Of the 22 Boronic acid positive isolates only five were detected by the MHT, and only one was detected by PCR assay as positive for KPC. When the 21 isolates that were negative by *bla*KPC PCR were tested for AmpC genes (DHA, EBC, MOX, FOX, CIT, and ACC), only two isolates turned out positive, both of which were *E*. *coli*. Out of f 20 MHT positive isolates, seven were positive for OXA-48 and only two positive for KPC by PCR. Out of the seven isolates detected by EDTA test as positive for metallo-β-lactamases, six were positive by PCR. This test was therefore more efficient at detecting activity of metallo-β-lactamases. The EDTA test, however, showed a strong association with detection of IMP type (*P* = 0.000) and NDM-1(*P* = 0.045) carbapenemases compared to VIM type metallo-β-lactamases (*P* > 0.05).

## Discussion

Resistance to the most commonly used antibiotics has greatly increased among gram-negative bacteria, especially in the family enterobacteriaceae, due to acquisition of genes producing ESBLs [3]. ESBLs are associated with co-resistance to other antibacterial classes thus organisms harboring them become resistant to multiple antibiotics hence limiting treatment options. Carbapenems are the antibiotics of choice for treatment of infections caused by ESBL producing bacteria [2,6]. However several studies have reported worldwide increased production of β-lactamases which hydrolyse all β-lactam antibiotics including carbapenems [2,5,6]. This study has revealed a prevalence of the carbapenamase phenotype of 22.4% and genotype of 28.6% among Enterobacteriaceae resistant to third generation cephalosporins. Our phenotypic prevalence was very high compared to 2.8% observed in Morocco using MHT screening [20]. The difference in these findings could be because the data from Morocco came from reported epidemic outbreaks of infection due to carbapenem-resistant *Klebsiella* spp. and *E*.*coli* strains in a hospital setting. Our prevalence is also much higher than that obtained in studies from China and Germany [21,22], as well as in a surveillance study in Spain which reported carbapenemase-encoding gene prevalence of 0.04% [23]. These differences may be due to restricted use of antibiotics in these countries compared to Uganda where most drugs are available over the counter without prescription by a clinician [24]. Our findings are however comparable to those observed in Nigerian, where a study reported a prevalence of 33.5% [25] in a hospital setting. However, that study only determined the prevalence of metallo-β-lactamases unlike ours which considered all carbapenemase classes. Furthermore, the genotypic prevalence seen in our study is comparable to observations in neighboring Tanzania that found it at 35% [5], probably due to similar antibiotics use patterns in both countries. For example in Tanzania antibiotic usage was noted to be at 85% in children under 5 years [26] while in Uganda it was at 43.2% in adults visiting health centres and community pharmacies [24]. These observations present a worrying trend of antimicrobial resistance in the East African region. Elsewhere, our genotypic findings are also comparable with those recorded by the European Antimicrobial Resistance Surveillance Network data for Greece where a prevalence of 43.5% was noted [20].

The most prevalent gene among the 196 study isolates was *bla*VIM at 10.7%., only a half of what was seen in neighboring Tanzania, where IMP types were the most predominant at 21.6% of 227 isolates studied [5]. However, most of the *bla*VIM, *bla*IMP, *bla*NDM-1, *bla*KPC and *bla*OXA-48 genes detected by PCR assay were negative phenotypically, suggesting that these isolates possessed the genes that may not have been expressed. A previous study suggested that beta-lactamases by gram-negative organisms are usually secreted especially when antibiotics are present in the environment [27], probably explaining the observation of unexpressed genes in our strain collection.

We detected a low prevalence of the *bla*NDM-1 gene (2.6%) among the study strains. The NDM-1 encoding gene is located on different large plasmids that are easily transferable to susceptible bacteria at a high frequency, and these plasmids are thought to be responsible for resistance to almost all antibiotics [28]. The prevalence of *bla*NDM-1 gene observed in our study is in agreement with observations in Tanzania, where it was seen in 3.1% of 227 isolates [5]. furthermore, the *bla*NDM-1 gene co-existed in the same isolate with at least two other carbapenamase genes in three of five isolates among which it was detected, probably explaining why these isolates were multidrug resistant. We also observed that urine and pus swabs had the largest number of bacteria carrying carbapenemase-encoding genes. These finding are similar to those observed in Tanzania and Nigeria [5,25]. This may be due to prolonged exposure of bacteria in the normal flora of the patients to antibiotics in these settings, hence an increased population of resistant strains [29]. The resistant organisms may have subsequently caused infection in the urinary tract and wounds. These findings however, are contrary to those obtained by a study in Indonesia [6] in which sputum was the sample with isolates with the highest carriage of these genes.

Antibiotic resistance results showed that out of 106 study isolates with temocillin diameters of ≥12mm and piperacillin/tazobactam diameters ≥16 mm, 98 (92.5%) were negative for *bla*OXA-48 gene assayed by PCR. This is slightly different from observations in the study done at National Reference Laboratories in Belgium and France [15] where a combination of temocillin diameters of ≥12mm and piperacillin/tazobactam diameters ≥16 mm had a negative predictive value of 99.2% for detection of OXA-48 carbapenemases. We also noted that only 25 of 36 (69.4%) isolates of temocillin zone diameter <12mm did not have *bla*OXA-48 gene, also lower than observations from the Belgium and France study where temocillin diameters, 12 mm alone had specificity of 90.0% [15].

When we compared the performance of the phenotypic tests to results obtained by PCR, of the 22 isolates detected by Boronic acid inhibitor based test, only one turned out positive by PCR. When the 21 isolates that were negative by blaKPC PCR were tested for AmpC genes (DHA, EBC, MOX, FOX, CIT, and ACC), only two isolates turned out positive. A possible explanation for the absence of KPC and ampC in the 19 isolates could be that Boronic acid also inhibits and detects other class A carbapenemases (other than KPC) such as Sme, IMI, NMC-A, and GES as observed elsewhere [30], which were not assayed for in this study. It was further noted that of the 20 MHT positive Isolates, only two were positive for *bla*KPC and Seven for *bla*OXA-48 by PCR. This low detection rate may be due to low-level carbapenem hydrolysis by ESBLs, particularly those of the CTX-M type coupled with porin loss as suggested elsewhere [31,32]. MHT was however, strongly associated with detection of OXA-48 than KPC β-lactamases. This is supported by observations elsewhere, that MHT has a good sensitivity for detecting OXA-48 β-lactamases [33]. Furthermore, out of seven isolates detected by the EDTA inhibitor assay, six were positive by PCR. This result is in agreement with observations in a study by Khosravi. *et al*, in which 100% of isolates detected by combined disk of carbapenem and EDTA were also positive by PCR [34]. However, Khosravi’s study also observed a low specificity of 43.1% just like in another study where most of the metallo-β-lactamase detected by PCR were missed by EDTA test.

This study has demonstrated a high prevalence of carbapenemases and carbapenem-resistance encoding genes in enterobacteriaceae isolated from patients at the National Referral Hospital in Uganda. All the five genes assayed (*bla*NDM-1, *bla*VIM, *bla*IMP, *bla*KPC and *bla*OXA-48) were detected in the study sample. The most prevalent gene was *bla*VIM and the least was *bla*NDM-1. In this study, almost a quarter of the isolates tested were phenotypically positive for carbepenemase activity. We therefore recommend that isolates be phenotypically tested if resistant to two third generation cephalosporins so as to inform patient care.
